# Serum Cortisol as a Biomarker of Severe Dengue

**DOI:** 10.3390/tropicalmed8030146

**Published:** 2023-02-27

**Authors:** Chansuda Bongsebandhu-phubhakdi, Vichit Supornsilchai, Suphab Aroonparkmongkol, Umaporn Limothai, Sasipha Tachaboon, Janejira Dinhuzen, Watchadaporn Chaisuriyong, Supachoke Trongkamolchai, Mananya Wanpaisitkul, Chatchai Chulapornsiri, Anongrat Tiawilai, Thawat Tiawilai, Terapong Tantawichien, Usa Thisyakorn, Nattachai Srisawat

**Affiliations:** 1 Division of Endocrinology, Department of Pediatrics, Faculty of Medicine, King Chulalongkorn Memorial Hospital, Chulalongkorn University, Bangkok 10330, Thailand; 2 Excellence Center for Critical Care Nephrology, King Chulalongkorn Memorial Hospital, Bangkok 10330, Thailand; 3 Center of Excellence in Critical Care Nephrology, Faculty of Medicine, Chulalongkorn University, Bangkok 10330, Thailand; 4 Tropical Medicine Cluster, Chulalongkorn University, Bangkok 10330, Thailand; 5 Banpong Hospital, Ratchaburi 70110, Thailand; 6 Photharam Hospital, Ratchaburi 70120, Thailand; 7 Division of Infectious Diseases, Department of Medicine, Faculty of Medicine, King Chulalongkorn Memorial Hospital, Chulalongkorn University, Bangkok 10330, Thailand; 8 Division of Nephrology, Department of Medicine, Faculty of Medicine, King Chulalongkorn Memorial Hospital, Chulalongkorn University, Bangkok 10330, Thailand; 9 Academy of Science, The Royal Society of Thailand, Bangkok 10300, Thailand

**Keywords:** dengue, severe dengue, biomarkers, cortisol, Thailand

## Abstract

Dengue infection presents a wide range of clinical symptoms. Serum cortisol is known as a severity predictor of serious infection but is not yet clearly understood in dengue infection. We aimed to investigate the pattern of cortisol response after dengue infection and evaluate the possibility of using serum cortisol as the biomarker to predict the severity of dengue infection. This prospective study was conducted in Thailand during 2018. Serum cortisol and other laboratory tests were collected at four time points: day 1 at hospital admission, day 3, day of defervescence (DFV) (4–7 days post-fever onset), and day of discharge (DC). The study recruited 265 patients (median age (IQR) 17 (13, 27.5)). Approximately 10% presented severe dengue infection. Serum cortisol levels were highest on the day of admission and day 3. The best cut-off value of serum cortisol level for predicting severe dengue was 18.2 mcg/dL with an AUC of 0.62 (95% CI, 0.51, 0.74). The sensitivity, specificity, PPV and NPV were 65.4, 62.3, 16 and 94%, respectively. When we combined serum cortisol with persistent vomiting and day of fever, the AUC increased to 0.76. In summary, serum cortisol at day of admission was likely to be associated with dengue severity. Further studies may focus on the possibility of using serum cortisol as one of the biomarkers for dengue severity.

## 1. Introduction

Dengue is a mosquito-borne viral disease that has spread to many regions of the world, particularly in Asian, African and Latin American countries, where it is endemic Dengue infection is one of the leading causes of serious illness and death among children and adults in these regions. In 2019, 5.2 million dengue cases were reported to the WHO (World Health Organization), with reported deaths increasing from the previous decade [1].

While many dengue infections produce only mild illness, it can cause a more serious acute flu-like illness. This can develop into severe infection with potentially fatal complications such as plasma leakage, fluid accumulation, respiratory distress, severe bleeding, or multiorgan impairment. Recently, the WHO determined warning signs to predict severe dengue infection, such as severe abdominal pain, persistent vomiting, rapid breathing, bleeding gums/nose, liver enlargement, restlessness and blood in vomit/stool [1,2]. Patients with warning signs may not necessarily develop severe dengue, while those without warning signs remain susceptible to developing a severe dengue infection. A previous study among the adult cohort found that no single warning sign alone or combined had a sensitivity to predict severe dengue of more than 64% [3].

The mechanisms and physiologic changes, especially in the endocrine system, which increase the risk of developing a severe dengue infection are poorly understood. The kinetics of endocrine changes have been widely studied in several critical illnesses, such as septic and cardiogenic shock, but have rarely been studied in dengue infection. Previous studies, in 1995, demonstrated that the highest mean serum cortisol level was found in the febrile stage of children with both dengue hemorrhagic fever (DHF) and dengue fever (DF). However, the mean cortisol level in DHF was higher than in DF. The serum cortisol levels subsequently decreased by half during the convalescent phase [4]. Another study revealed that serum cortisol levels tended to be insufficient during the course of dengue infection, both during the febrile stage in children with DF and the shock stage in persons with DHF [5]. In 2019, a study of adults showed that patients with DHF had slightly higher median cortisol concentrations than patients with DF in febrile phase, but not on the day of defervescence or later [6].

To the best of our knowledge, there have been no substantial studies that demonstrated the changes in serum cortisol levels over time in individuals infected with dengue virus. Early cortisol response may predict the severity of dengue infection, which could potentially serve as one of the biomarkers for severe dengue disease. To address this knowledge gap, this study aimed to (1) investigate the pattern of cortisol response at different timepoints in patients with severe and non-severe dengue infections, and (2) evaluate the possibility of using cortisol levels at the day of hospital admission as a clinical biomarker to predict dengue severity.

## 2. Materials and Methods

### 2.1. Study Design and Setting

A prospective study was performed at two hospitals in central Thailand, including Banpong hospital (secondary care hospital, 420 beds) and Photharam hospital (secondary care hospital, 350 beds).

### 2.2. Study Population

All patients who were diagnosed with dengue infection at hospital admission in either the pediatric or medicine departments between 1 January and 30 June 2018 were recruited in the study. Patients were classified as having dengue based on the presence of IgM antibodies (Ab) or nonstructural protein 1 (NS1) or through the detection of genomic dengue RNA using RT-PCR. Samples were screened using a one-step immunochromatographic assay designed to detect both dengue virus NS1 antigen and antibodies (IgG/IgM) to the dengue virus (SD BIOLINE Dengue Duo kit, catalog number 11FK46, SD Bioline, Korea) according to the manufacturer’s instructions. Viral RNA was also extracted for the molecular detection of DENV [7]. Patients who were not proven to be infected through tests or had co-infection were excluded.

Informed consent was obtained from the patients or parents/guardians. The study was carried out according to the guidelines of the Declaration of Helsinki and Good Clinical Practice. The study protocol was approved by the Ethics Committees of Banpong Hospital (REC No. 009/2562) and Potharam Hospital (REC No. 32/2562). Blood samples from participants were collected at four time points, including on the day of enrollment, day 3, day of defervescence (4–7 days after the appearance of the fever), and day of discharge. The defervescence was determined when the body temperature returned to normal after high fever. The first blood sample was collected when the patients were admitted to the hospital, and other samples were collected in the early morning (8.00–9.00 AM). The laboratory was analyzed, and the serum and plasma samples were stored in aliquots at −80 °C until further analysis. We collected clinical and laboratory data on confirmed dengue cases from the hospital medical record system throughout the hospitalization period.

Patients were classified as either part of the severe dengue infection group or non-severe dengue infection group according to WHO criteria (2009) [8]. Severe dengue infection was defined by the following criteria: (1) severe plasma leakage, which leads to shock (dengue shock syndrome) or fluid accumulation with respiratory distress; (2) severe bleeding as evaluated by a clinician; and (3) severe organ involvement including elevated AST or ALT liver enzyme, impaired consciousness, or heart or other organ failure.

### 2.3. Measurement of Serum Cortisol

Serum cortisol was stored in aliquots at −80 °C and then analyzed in the central lab. Cortisol is a steroid hormone that is generally minimally affected by freeze–thaw cycles [9]. Serum cortisol was measured via MAGLUMI 600 chemiluminescence immunoassay (CLIA) (Snibe Diagnostics, Shenzhen, China) according to the manufacturer’s instructions.

### 2.4. Statistical Analysis

Statistical analysis was performed using SPSS Version 22 (SPSS, Chicago, IL, USA). Figures were drawn using GraphPad Prism 8 (GraphPad Software Inc., La Jolla, CA, USA). Categorical data were expressed as percentages and compared between groups using chi-squared testing. Continuous variables were presented as means ± standard deviation (SD) when data were normally distributed, and for non-normally distributed variables as median and interquartile range. Student’s t test and the Mann–Whitney test were used to analyze the differences between two continuous variables. The area under the receiver operating characteristic curve (AUC-ROC) was calculated to assess the diagnostic performance of serum cortisol level. In addition, multivariable logistic regression was utilized for examining the predictive ability of serum cortisol levels for dengue severity. *p*-values below 0.05 were considered statistically significant.

## 3. Results

A total of 376 patients were clinically diagnosed with dengue infection on the first day of admission. Using these serologic results, 111 patients were excluded from the study. A total of 26 patients (9.8%) of the remaining 265 patients were diagnosed with severe dengue infection. The median day of fever that patients were admitted to the hospital was 3 days (IQR 2,4) in the non-severe dengue group and 3 days (IQR 1,4) in the severe dengue group. The percentage of patients who were positive for the NS1 antigen, IgM and IgG, was 72.9, 55.8 and 59.6%, respectively. (Table 1) No one died during the study period. A total of 120 patients (45.3%) were male. The age range and median age of the patient (IQR) were 7–68 and 17 years (13, 27.5), respectively. The median age of the severe and non-severe groups was 13.5 and 18 years, respectively.

In the group of severe dengue (n = 26), twenty-two patients were diagnosed with dengue shock syndrome, one patient with shock and acute kidney failure, one patient with acute kidney failure, one patient with shock and acute liver failure, and one patient with acute liver failure.

The common clinical symptoms found in both groups were having a history of vomiting of more than two times (18%) and abdominal pain (17%). The median body temperature, pulse rate, respiratory rate, and the mean (SD) neutrophil percentage in the severe group was significantly higher than the non-severe (Table 1).

### 3.1. Serum Cortisol Level

**Figure 1:**
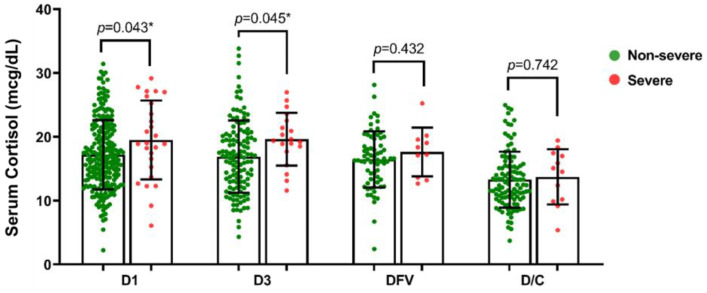
Serum cortisol (mcg/dL) during admission in non-severe and severe dengue infection. DFV: day of defervescence, D/C: day of discharge from hospital. * *p*-value < 0.05.

**Figure 2:**
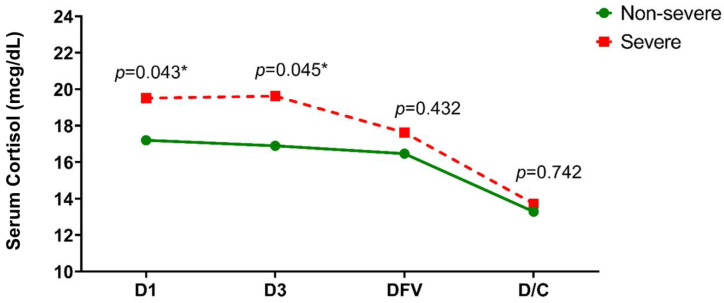
Mean of serum cortisol (mcg/dL) during admission. DFV: day of defervescence, D/C: day of discharge from hospital. * *p*-value < 0.05.

Serum cortisol levels at different time points of admission were compared between the severe and non-severe dengue infection patients. On day 1, cortisol concentration in the severe group was significantly higher than in the non-severe group (19.5 ± 6.2 vs. 17.2 ± 5.4, *p* = 0.043). At day 3, patients with severe dengue infection had significantly higher cortisol concentrations than that of the non-severe group (19.6 ± 4.1 vs. 16.8 ± 5.7, *p* = 0.045). At DFV and the day of discharge, the mean cortisol levels showed no difference between the groups (Figure 1). The pattern of the mean cortisol concentrations at all timepoints was also similar among both groups. (Figure 2) Mean cortisol concentrations were constantly high during the first and third day of the hospital stay and then gradually decreased by the defervescence day and the day of discharge.

### 3.2. Serum Cortisol Levels as a Biomarker to Predict Severity of Dengue Infections

**Figure 3:**
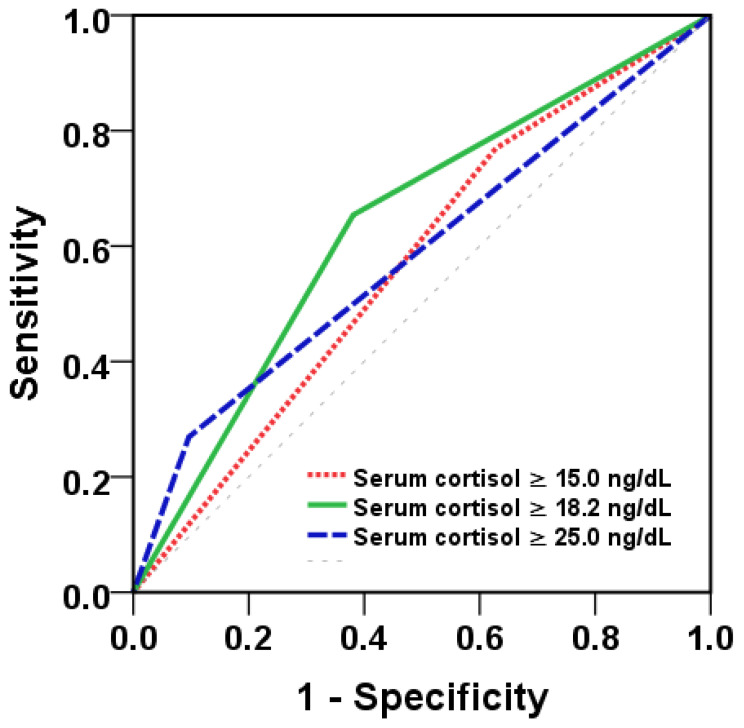
The area under the curve (AUC) to predict the severity of dengue infection.

Table 2 shows the derived sensitivities, specificities, positive predictive values, and negative predictive values for cortisol concentration levels at the first day of admission and displays the maximum summation of sensitivity and specificity. We determined the most appropriate cutoff of cortisol concentration at the first day of visit that could best predict dengue severity by ROC analysis. The best cut-off value for diagnosis of severe dengue was 18.2 (mcg/dL) with AUC of 0.62 (95% CI, 0.51, 0.74). The sensitivity, specificity, PPV and NPV were 65.4, 62.3, 16 and 94%, respectively. (Table 2 and Figure 3).

Multiple regression analysis of the baseline variables was run to predict severe dengue infection. The odds ratio (OR, 95% Confidence interval, CI) from the multivariate analysis of persistent vomiting, day of fever, and serum cortisol at day 1 of visit (<18.2 and ≥18.2 mcg/dL) were 3.68 (95% CI, 1.42, 9.56), 0.82 (95% CI, 0.59, 1.12) and 2.15 (95% CI, 0.87, 5.54), respectively (Table 3).

Based on the regression analysis, we combined cortisol concentration at day 1 of visit with the other clinical or laboratory data to improve the prediction model for severe dengue. The AUC of cortisol concentration at day 1, persistent vomiting, and day of fever was increased to 0.74 (*p* < 0.001) (Table 4).

## 4. Discussion

In this prospective study, we demonstrated changes in serum cortisol levels in patients with dengue infection at different timepoints during hospitalization. Cortisol levels rose in response to the stress of illness from the first day of admission, and then declined by the day of hospital discharge. (Figure 1) The trend of cortisol level changes in individuals with non-severe dengue infection was similar to those with severe dengue infection (Figure 1). However, patients with severe dengue infection were more likely to have higher cortisol levels than non-severe patients. Elevated cortisol concentrations have been widely described as an important sepsis biomarker in septic shock due to Gram-positive and Gram-negative sepsis. It seems probable that the higher cortisol levels may be indicative of a more severe clinical situation [10,11]. During sepsis, the surging of serum cortisol levels reflects the adaptive hormonal response of the hypothalamic–pituitary–adrenal (HPA) axis, which is important for catecholamine effects and vasopressin release. Cortisol itself also has an important role in maintaining the vascular response to catecholamines, leading to the maintenance of an adequate blood perfusion pressure in vital organs [10,11].

Our study demonstrated higher levels of cortisol in severe dengue than those in non-severe dengue. This outcome was relatively contrary to the previous study conducted by Phung NTN et al. (2021) which suggested that cortisol levels of children with severe DSS were lower than those of children with non-severe DSS [12]. There are two possible explanations for this conflicting result. First, our study focused on all ranges of severe dengue infection classified using the recent WHO 2009 criteria, including severe plasma leakage, bleeding manifestation, and severe organ injury, whereas the previous study evaluated serum cortisol only in patients with severe plasma leakage (DSS), using the WHO 1997 criteria. Second, our study measured cortisol levels on the first day of admission, but Phung NTN et al. measured them on the shock recognition period. Severe DSS may be linked to adrenal dysfunction during the shock period. In our study with a large number of participants, we had only two patients with severe dengue shock syndrome (systolic blood pressure less than 70 mmHg). To understand the dynamic of adrenal function during dengue shock syndrome, we suggest that the further study should focus on adrenal function over the course of the disease.

As compared to the other common viral infections such as influenza and COVID-19, the cortisol levels increased after being infected. However, the cortisol levels in viral infections were not as high as levels found in bacterial sepsis, which were comparable with the cortisol concentrations reported in our study. The postulated mechanism is that the virus expressed some amino acid sequences that had molecular mimics to endogenous adrenocorticotropin hormone (ACTH) in the host and subsequently produced antibodies against the amino acid sequences causing low ACTH and adrenocortical insufficiency [13,14,15]. Autopsy studies have also suggested a direct cytopathic effect of the virus [13,14]. Regarding dengue infection, the literature is scarce. No previous studies have demonstrated an immune response causing dysregulation of the HPA axis after dengue infection. There was only one study in guinea pigs showing that the adrenal glands of dengue-infected animals were occasionally markedly enlarged but had not shown degeneration or necrosis of the adrenal cortical cells [16]. The direct effect of dengue on adrenal glands and the pathophysiologic response of the HPA axis to severe dengue infection should be further investigated.

Interestingly, our results revealed that cortisol was declining in the defervescence stage compared to the fever stage in both the severe and non-severe dengue groups. The pattern found here corresponds to that of Sura-Amornkul S [6]. This decline might be due to molecular mimics and/or the direct effect of the virus on the adrenal glands, as we discussed above. In the severe dengue group, we found that serum cortisol decreased during the defervescence stage, even in hemodynamic instability. This may contribute to worse clinical outcomes. Additional studies are needed to understand the cause of this declining cortisol.

One important finding in the present study showed that the cortisol levels in the severe dengue group were higher than those in the non-severe dengue group at the first day of visit. Therefore, we might use cortisol levels as a potential biomarker for severity of dengue infection. Elevated levels of cortisol (more than 18.2 mcg/dL) were possibly able to predict dengue severity with a sensitivity of 65.4% and specificity of 62.3%. The predictive value of serum cortisol did improve to 72% when it was combined with clinical model factors, including the day of fever and persistent vomiting (Figure 3).

Hospital admissions due to dengue are considered by physicians based on a patient’s condition such as the degree of dehydration and/or warning signs of dengue severity, including abdominal pain, persistent vomiting, clinical fluid accumulation, mucosal bleed, lethargy, restlessness, liver enlargement, or increasing hematocrit with a decreasing platelet count. These patients often progress to severe dengue. Adding cortisol level as a factor with the current clinical model of severity predictors may facilitate improved care regarding severe complication surveillance.

Previous studies have noted immune activation markers including number of immune cells, increased levels of cytokines, chemokines and complement antibodies, and other soluble factors such as severity biomarkers [17]. Hematologic parameters such as D-dimer have also been proposed for predicting dengue severity [18,19]. Genetic susceptibility for severe dengue has also been suggested [20,21]. However, these parameters require highly complex laboratory testing and may not be applicable in general hospital settings. Serum cortisol levels are more available and feasible as a biomarker of dengue severity in clinical practice. Further research should be undertaken to validate the efficacy of cortisol together with clinical model factors to predict severity in dengue infection.

This study had several strengths. First, to our knowledge, this study had one of the largest sample sizes with the broadest age range of patients (children and adult) compared to previously published studies investigating the association between the levels of cortisol and severe dengue infection. Second, we used the 2009 revised dengue severity classification, which we believe is more suitable than the WHO 1997 classification, because it better covers the entire spectrum of severe dengue infection [22]. Third, this study has examined the changes of cortisol from hospital admission until discharge. Measurements at different time points may help us to better understand changes in cortisol levels during the development of dengue illness.

This study was not without limitations. First, some of our patients were diagnosed with dengue infection through presence of IgM antibodies (Ab) or nonstructural protein 1 (NS1), which may show cross-reactivity between different flaviviruses. Second, we have a low number of cases with organ impairment such as acute kidney injury or acute liver injury. Further studies are needed to determine whether our results are valid for these types of severe dengue infection complications. Third, we have tested the role of serum cortisol in predicting severe dengue infection at only a single time point (on the first day of hospital admission) and could not answer the predictive value of cortisol at other time points. Nevertheless, risk stratification and prognostication using a biomarker is likely to be useful only when biomarker concentrations are measured early. Fourth, our study enrolled only Thai patients with dengue infection. The differential expression of cortisol should be studied in an extended cohort with diverse populations to validate the finding in this study.

To develop a full picture of the response of the HPA axis to dengue infection, additional studies with more severe dengue infections are needed to assess whether the adrenocortical response in severe dengue infection is sufficient. Randomized controlled trials of early use of corticosteroids in possible cases of severe dengue may also elicit a beneficial effect on the morbidity and mortality of severe dengue infection.

## Figures and Tables

**Table 1:** Patient demographic and disease characteristics at admission.

	Total n = 265 (100%)	Non-Severe n = 239 (90.2%)	Severe n = 26 (9.8%)	*p*-Value
Male, n (%)	120 (45.3%)	107 (44.7%)	13 (50%)	0.681
Age (years), median (IQR)	17 (13,27.5)	18 (14.0,28.0)	13.50 (10.8,24.0)	0.061
Day of fever (day), median (IQR) *	3 (2, 4)	3 (2, 4)	3 (1, 4)	0.015
**Pertinent symptoms**				
History of vomiting (>2 times) *, n (%)	48 (18.1%)	38 (15.9%)	10 (38.5%)	0.012
Abdominal pain, n (%)	45 (17%)	40 (16.7%)	5 (19.2%)	0.783
Drowsiness, n (%)	4 (1.5%)	3 (1.3%)	1 (3.8%)	0.340
Mucosal bleeding/GI bleeding, n (%)	23 (8.7%)	20 (8.4%)	3 (11.5%)	0.481
**Physical Examination**				
Body temperature (°C) median (IQR) *	37.7 (36.9, 38.8)	37.6 (36.8, 38.7)	38.7 (37.7, 39.8)	0.001
Systolic blood pressure (mmHg), median (IQR)	110 (100, 120)	110 (100, 120)	110 (100, 120)	0.822
Diastolic blood pressure (mmHg), median (IQR)	70 (60, 70)	70 (60, 70)	70 (60, 80)	0.140
Pulse rate (/min), median (IQR) *	94 (82, 105)	92 (80, 104)	110 (100, 115.50)	<0.0001
Respiratory rate (/min), median (IQR) *	20 (20, 24)	20 (20, 24)	24 (20, 28)	0.001
**Laboratory findings**				
WBC (10^3^/cumm), median (IQR)	3.20 (2.30, 4.65)	3.22 (2.30, 4.69)	3.11 (2.64, 4.45)	0.298
Neutrophil (%), mean (SD) *	51.41 (18.40)	50.25 (18.54)	61.96 (13.16)	0.002
Hb (gm%), mean (SD)	13.33 (1.81)	13.36 (1.80)	13.17 (1.95)	0.627
Hct (%), mean (SD)	40.16 (5.00)	40.24 (4.86)	39.41 (6.15)	0.420
Platelet count 10^3^/cu.mm, median (IQR)	96.00 (64.00, 148.75)	96.00 (64.00–146.25)	105.00 (63.25, 156.75)	0.285
NS1 Ag: Positive, n (%) *	186 (72.9%)	162 (70.4%)	24 (96%)	0.004
IgM: Positive, n (%) *	148 (55.8%)	142 (59.4%)	6 (23.1%)	0.001
IgG: Positive, n (%) *	158 (59.6%)	148 (61.9%)	10 (38.5%)	0.034

* p-value < 0.05.

**Table 2:** Serum cortisol at various cutoff value to predict dengue severity.

Cortisol Cutoff (mcg/dL)	Sensitivity	Specificity	PPV	NPV
15.0	0.77	0.37	0.12	0.94
18.2	0.65	0.62	0.16	0.94
25.0	0.27	0.90	0.23	0.92

**Table 3:** Multiple regression analysis of baseline variables in predicting severe dengue.

Variables	Univariate			Multivariate		
	OR	95% CI	*p*-Value	OR	95% CI	*p*-Value
Demographic profile						
Age	0.97	0.93–1.01	0.181			
Gender	0.82	0.36–1.84	0.624			
Fever details						
Day of fever	0.70	0.53–0.93	0.015 *	0.82	0.59–1.12	0.211
Warning signs						
Abdominal pain or tenderness	1.18	0.42–3.33	0.748			
Persistent vomiting	3.31	1.40–7.83	0.007 *	3.68	1.42–9.56	0.008 *
Mucosal bleed	1.43	0.39–5.18	0.587			
Lethargy, restlessness	3.15	0.32–31.40	0.329			
Liver enlargement	0.00	0.000	-			
Lab values on admission						
White blood cell	1.00	1.00–1.00	0.294			
Neutrophil	1.04	1.01–1.06	0.003 *	1.08	1.00–1.17	0.058
Lymphocyte	0.97	0.94–1.00	0.029 *	1.07	0.97–1.17	0.191
Platelets	1.00	1.00–1.00	0.131			
Hemoglobin	0.95	0.76–1.18	0.626			
Hematocrit	0.97	0.89–1.05	0.418			
Aspartate transaminase (AST)	1.00	1.00–1.00	0.102			
Alanine aminotransferase (ALT)	1.00	1.00–1.01	0.422			
Serum cortisol level (<18.2 vs. ≥18.2 mcg/dL)	3.13	1.34–7.31	0.009 *	2.15	0.87–5.34	0.098

* p-value < 0.05.

**Table 4:** Combination of biomarkers to maximize the area under the ROC curve.

Variable	AUC	*p*-Value
Cortisol	0.62	0.038 *
Persistent vomiting	0.61	0.055
Day of fever	0.36	0.017 *
Persistent vomiting + Day of fever	0.69	0.010 *
Persistent vomiting + Cortisol	0.71	0.001 *
Day of fever + Cortisol	0.67	0.005 *
Day of fever + Persistent vomiting + Cortisol	0.74	<0.001 *

* *p*-value < 0.05.

## Data Availability

The datasets used and/or analyzed during the current study are available from the corresponding author on reasonable request.
